# Subjective memory complaints in people with epilepsy: Are there “signature” complaints associated with anxiety and depression?

**DOI:** 10.1002/epi4.70027

**Published:** 2025-03-22

**Authors:** Cassandra Trend, Isha Puntambekar, Sallie Baxendale

**Affiliations:** ^1^ University College Hospital London UK; ^2^ Department of Clinical and Experimental Epilepsy UCL Queen Square Institute of Neurology London UK

**Keywords:** anxiety, depression, epilepsy, mood, subjective memory complaints

## Abstract

**Objective:**

While there is a relatively weak association between cognitive complaints and performance on standardized tests of memory function, elevated levels of depression and anxiety are highly correlated with subjective memory complaints in people with epilepsy (PWE). The study examined whether there are “signature” constellations of memory complaints that are associated with anxiety and depression in PWE. If identified, these signatures may alert clinicians to the likelihood of mood playing a role when presented with these complaints in the neurology clinic.

**Methods:**

Three hundred and seventy‐five adults with epilepsy, mean age 37 (s.d. 12.8), completed a Subjective Memory Questionnaire (SMQ), rating how often they experienced 19 different types of memory difficulty. Frequencies ranged from never to more than once a day on a six‐point scale. They also completed the Hospital Anxiety and Depression Scale.

**Results:**

A principal component analysis of responses on the SMQ revealed three primary factors. Factor 1 comprised items primarily related to verbal memory lapses in social settings such as forgetting people's names, repetition and rambling in conversation, and difficulties following the thread of a discussion. Factor 2 comprised items related to losses from the core store of memories such as failure to recognize close relatives, getting lost, and forgetting autobiographical details. Factor 3 related to organizational/attentional aspects of memory with an executive component. People who reported moderate/severe levels of anxiety and depression on the HADS reported a higher frequency of memory failures in the social domain than those with no mood disturbance. Anxiety was associated with memory complaints mediated by executive functions, while depression was associated with increased reports of losses from the core memory store.

**Significance:**

Anxiety and depression are associated with different subjective memory complaints in people with epilepsy. Paying attention to the nature of these complaints may help in the management of these difficulties.

**Plain Language Summary:**

Anxiety and depression are associated with different patterns of memory complaints in people with epilepsy. In this study, we found that elevated levels of anxiety and depression are associated with memory complaints that impair social function. Anxiety is also associated with problems that have an executive basis, while depression is associated with reports of losses from the core memory store. Recognizing these patterns may help clinicians identify the most effective interventions for these difficulties.

**Key points**

1. Anxiety and depression are associated with different subjective memory complaints in people with epilepsy.
2. Elevated levels of anxiety and depression are associated with memory complaints that impair social function.
3. Anxiety is also associated with problems that have an executive basis.
4. Depression is associated with reports of losses from the core memory store.
5. Recognizing the potential influence of mood on memory complaints helps clinicians to tailor the most effective interventions.

## 1 INTRODUCTION

Cognitive concerns are commonly reported by people with epilepsy^1^ and can have a significant impact on quality of life, with reductions in access to employment and increased social exclusion.^2^ These cognitive complaints are diverse and multifactorial in origin and can result from epilepsy‐related factors,^3^ the impacts of treatments aimed at controlling seizures, and broader factors associated with the diagnosis of epilepsy, including elevated levels of anxiety and low mood.^4^ While specific difficulties with attention, executive function, and word finding are reported by people with epilepsy, memory impairment is the most frequent cognitive complaint reported in the epilepsy clinic.^5^, ^6^

Memory function is typically assessed with standardized tests in a clinical neuropsychological assessment. However, it is well established that the association between subjective cognitive complaints and performance on formal tests of memory function is weak.^7^, ^8^, ^9^, ^10^, ^11^ This is due to a number of factors. The mismatch between memory complaints and function on formal tests may reflect the structure of the test. There is no single test that covers all aspects of memory function. Proficiency in different domains of memory function (e.g., encoding, consolidation, retrieval, susceptibility to distraction etc.) can and do dissociate.^12^ Studies from Europe and Australia indicate that at least part of the disconnect between subjective memory complaints and performance on standardized tests of memory function may also be due to the failure of the latter to assess long‐term forgetting^13^ and associated aspects of autobiographical memory.^14^ Extending retention intervals of 4 weeks on a verbal learning task leads to better correspondence of subjective with objective memory impairments, questioning the ecological validity of short‐term memory tests and emphasizing the fact that these memory complaints may no longer be as “subjective” as once believed.^13^

Other reasons for the weak association may include differences in the way language is used by people with epilepsy and professionals when discussing “memory” difficulties. Word‐finding difficulties are one of the most common examples given by people with epilepsy reporting everyday memory difficulties, but these are not typically assessed using standardized memory tests. Thus, difficulties attributed to “memory” by people with epilepsy may be more closely related to performance on formal tests of function in other cognitive domains, such as attention, concentration, expressive language (word finding difficulties), or executive function.^1^

While there is a relatively weak association between memory complaints and performance on standardized tests of memory function, low mood, depression, and elevated levels of anxiety are highly correlated with subjective complaints in this population.^7^, ^15^, ^16^, ^17^, ^18^, ^19^, ^20^, ^21^, ^22^

Understanding the factors that contribute to subjective memory complaints is an important aspect of epilepsy management. Cognitive complaints that result from the side effects of anti‐seizure medication, underlying pathology, or low mood will all require a different management approach (medication adjustment, cognitive rehabilitation and treatment for a mood disorder, respectively). These complaints may also represent the preclinical phase of Alzheimer's Disease in some people with epilepsy. Ruggeri et al. argue that subjective memory decline is not actually “subjective,” but rather a specific and defined clinical entity in this subgroup of patients.^23^

While an expert neuropsychological assessment can help to identify the etiology of memory complaints, clinical neuropsychology is a scarce resource in much of the world^24^ and is not available to the majority of people with epilepsy. The aim of this study was to examine whether there are individual “signature” memory complaints, or constellations of memory complaints that are more strongly associated with anxiety and depression than others. If these signatures can be identified, the presence of these complaints in the clinic may alert clinicians to the likelihood of these factors playing a significant role when they are presented with these complaints in the neurology clinic.

## 2 METHODS

This study represents an analysis of the responses on the Hospital Anxiety and Depression Scale (HADS)^25^ and Subjective Memory Questionnaire (SMQ)^26^ which were administered as part of a routine clinical assessment to 375 consecutive adult patients attending our department for a neuropsychological assessment in 2023 (The National Hospital for Neurology & Neurosurgery, London and the UCLH Chalfont Centre for Epilepsy, United Kingdom). Participants were aged between 17 years and 80 years (Mean: 37 years, SD: 12.8); 198 were female, and 177 were male. Epilepsy diagnoses were based on expert consideration of clinical history with seizure semiology, neuroimaging findings, and electroencephalography (EEG). Patients diagnosed with non‐epileptic attack disorder or whose seizures were determined to be the result of functional neurological disorder were excluded from the analyses. Patients who had undergone epilepsy surgery were also excluded from the analyses, since factors associated with surgery can influence subjective memory complaints;^27^ however, those undergoing presurgical evaluation were included.

All patients were referred to our department within the National Health Service by other professionals, primarily neurologists, neuropsychiatrists, and general practitioners. Patients were referred for a variety of reasons, including as part of their diagnostic workup, the evaluation of cognitive complaints, and to establish a baseline prior to surgery or changes in medication. All patients were taking at least one anti‐seizure medication at the time of the neuropsychological assessment.

One hundred and four patients had temporal lobe epilepsy (*n* = 39 Right; *n* = 45 left, *n* = 20 unclear); the remaining 271 had extra‐temporal seizures (*n* = 46), generalized epilepsy (*n* = 51), or other/unclassified epilepsy (*n* = 174).

### 2.1 Measures

The HADS is a 14‐item scale yielding scores for anxiety (7 items) and depression (7 items). Each item is rated on a 4‐point Likert scale, resulting in a final score for anxiety ranging from 0 to 21 and a final score for depression ranging from 0 to 21. Higher scores represent higher levels of anxiety and depression. The cutoff points for the anxiety and depression subscales are as follows: 0–7 = normal; 8–10 = mild; 11–15 = moderate; and 16–21 = severe.

The subjective memory questionnaire (SMQ) is a 19‐item scale which presents 19 common memory problems such as forgetting to take your tablets, or forgetting someone's name (See Appendix A). The respondent is asked to rate how often each of the problems occur on a six‐point scale ranging from “never,” to “happens more than once a day.” Each item is scored from 1 to 6 with the higher number representing increased frequency of the problem. The sum of the 19 items provides an overall SMQ score. See Appendix A for the scoring criteria of the SMQ.

### 2.2 Ethical approval

Approval for this study was granted by the hospital approval board (Ref: UCLHDEX350) committee. All data were fully anonymized prior to analyses to ensure that the study conformed with local and national ethical guidelines for the analyses of routine data collected in clinical settings.

### 2.3 Statistical analyses

Analyses were conducted using SPSS. A factor analysis was used to identify the principal components of the Subjective Memory Questionnaire, with extraction based on eigenvalues >1. A varimax rotation was employed with a maximum of 25 iterations for convergence. Factor scores were saved using the Anderson‐Rubin method.

Patients scoring 8–10 on the HADS are classified as having mild levels of anxiety or depression on their respective scales. Patients scoring 11–14 have moderate anxiety/depression, while those with a score of 15 or above are classified as having severe levels of anxiety or depression. A cutoff score of 11 or above was used to identify those who reported moderate or severe levels of anxiety or depression on the respective subscales of the HADS. Independent *t*‐tests were used to compare the mean scores on each factor identified in the SMQ factor analyses for the depressed (scores of 11 or above) versus nondepressed, and anxious (scores of 11 or above) versus nonanxious groups, respectively.

## 3 RESULTS

The mean total score for the whole group on the SMQ was 58.9 (s.d. 20.3, range: 19–103). Patients who reported moderate to severe levels of anxiety and depression had significantly higher total scores on the SMQ than those who scored in the normal‐mild range on the respective HADS subscales, with large effect sizes: Anxiety *t* = −7.3, df 373, *p* < .001 Cohens *D* = 19.0: Depression *t* = −5.0, df 373, *p* < .001, Cohens *D* 19.7.

**FIGURE 1:**
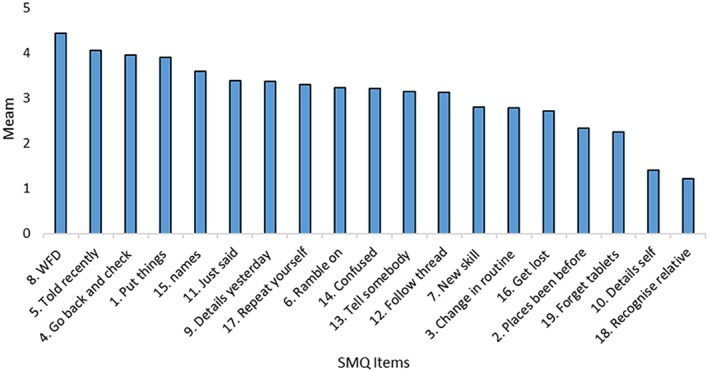
Frequency with which SMQ items are endorsed.

The Friedman test indicated significant differences in the level of endorsement across the items on the SMQ (Chi square 2246.0, df 18, *p* < .0001). Word‐finding difficulties were endorsed most frequently, while failure to recognize a close relative was endorsed least frequently. Figure 1 illustrates the relative endorsement of each of the items on the SMQ.

### 3.1 SMQ factor analysis

**FIGURE 2:**
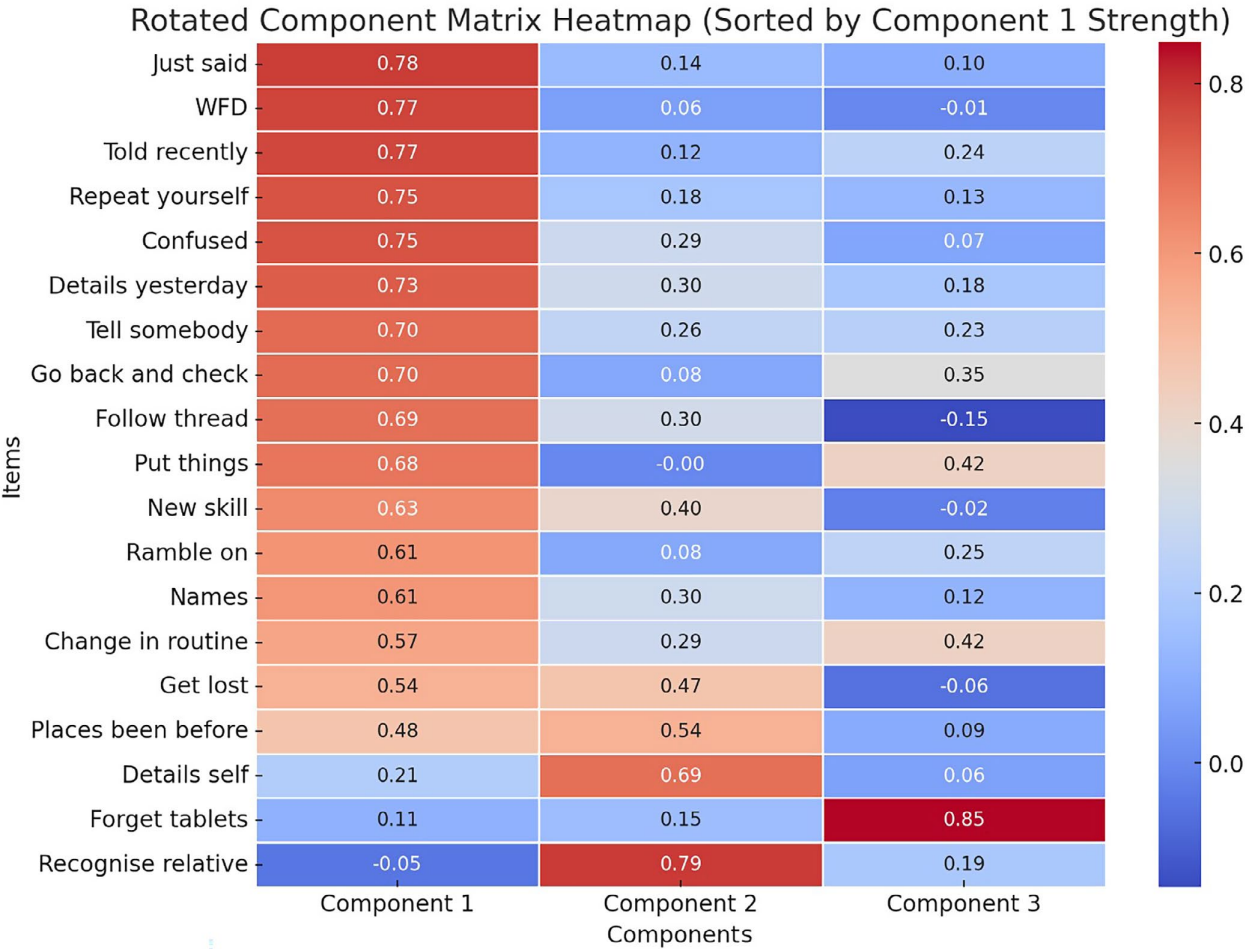
Rotated Component Matrix: Extraction Method, Principal Component Analysis, and Varimax with Kaiser Normalization. Rotation converged in six iterations. Component 1 “Social” refers to memory problems that impact social functions. Component 2 “Losses” refers to losses from the core memory store. Component 3 “Executive” refers to the executive aspects of memory function.

A factor analysis was conducted to explore the underlying structure of subjective memory experiences in individuals with epilepsy. The analysis included 19 variables related to memory experiences. The Kaiser–Meyer–Olkin measure of sampling adequacy was 0.950, indicating excellent sampling adequacy. Bartlett's test of sphericity was significant (χ^2^[171] = 3760.2, *p* < .001), supporting the factorability of the correlation matrix. Three factors were extracted using principal component analysis with varimax rotation with Kaiser Normalization, which accounted for 59.58% of the total variance in the data. The factor loadings revealed distinct patterns of association between the items and factors. See Figure 2.

Factor 1 included variables primarily related to verbal memory lapses in a social setting and included forgetting people's names, forgetting information that you have recently been told, repeating yourself and rambling on in conversation and having difficulties following the thread of a discussion. We named this factor “Memory problems that impact social function” (Social). Factor 2 comprised of variables that related to losses from the core store of memories; failure to recognize relatives, getting lost, forgetting core autobiographical details and failing to recognize places that you have visited before. We named this factor “Losses from the core store” (Losses). The third factor appeared to relate to organizational/attentional aspects of memory with an executive component and consisted of the items related to forgetting where you have put things, forgetting changes in routine and forgetting to take tablets. We named this factor “Executive aspects of memory function.”

### 3.2 Mood and Subjective Memory Complaints

#### 3.2.1 Anxiety

**FIGURE 3:**
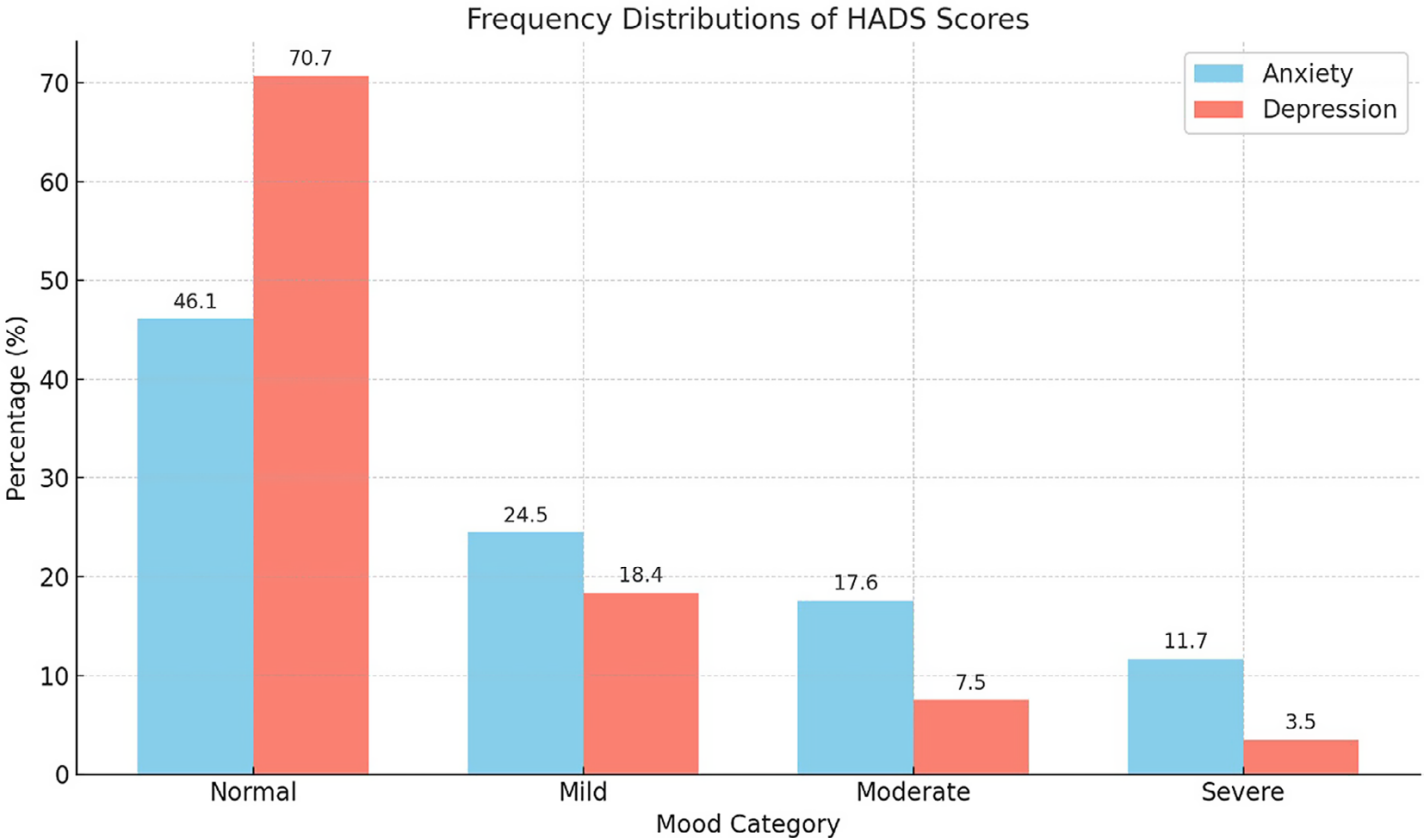
Frequency distributions of scores on the anxiety and depression subscales of the HADS.

Distributions of scores on the anxiety and depression subscales of the HADS are presented in Figure 3. One hundred and ten of the sample (29.3%) reported moderate or severe levels of anxiety. There was a significant difference between the two groups (anxious/not anxious) on Factor 1 (Social) and Factor 3 (Executive) with a large effect size for both factors (Social *t* = −6.1, df 373, *p* < .001, Cohen's *d* = 0.95; Executive *t* = −3.5, df 373, *p* < .001 Cohen's *d* = 0.99). Patients who were moderately or severely anxious reported problems with the items comprising these factors significantly more often than patients who did not report significant levels of anxiety. The two groups did not differ in their endorsement of the items that comprised Factor 2 (Losses *t* = −1.58, df 161, *p* > .05).

#### 3.2.2 Depression

Forty‐one of the sample (11%) reported moderate or severe levels of depression on the HADS. There was a significant difference between the two groups (depressed/not depressed) on Factor 1 (Social) and Factor 2 (Losses) with a large effect size for both factors (Social; *t* = −4.4, df 373, *p* < .001, Cohen's *d* = 0.97: Losses; *t* = −1.9, df = 45.1, *p* < .05, Cohen's *d* = 0.99). Patients who were moderately or severely depressed reported problems with the items comprising these factors significantly more often than patients who did not report significant levels of depression. The two groups did not differ in their endorsement of the items that comprised Factor 3 (Executive; *t* = −0.33, df 46.1, *p* > .05).

### 3.3 Summary of analyses

Our findings indicate that patients with high levels of anxiety and depression differ in both the nature and extent of subject memory complaints. Elevated levels of anxiety and depression are associated with greater endorsement of memory problems that impact social function. High levels of anxiety are also associated with memory complaints that have an executive basis, but not losses from their core memory store. Conversely, patients who report moderate to severe levels of depression report greater losses from their core memory store, but do not differ in reports of difficulties associated with executive function.

## 4 DISCUSSION

We explored whether “signature” subjective memory complaints could be identified in patients with elevated levels of anxiety and depression. Consistent with previous literature, we found a relationship between the extent of mood disturbance and subjective memory complaints. However, this relationship is nuanced, and some complaints are more common in anxiety than depression and vice versa. A factor analysis revealed three principal components of subjective memory complaints in our sample: memory problems that impact social function, losses from the core store, and those related to executive dysfunction. While memory complaints that impact social function were associated with elevated levels of both anxiety and depression, complaints related to losses from the core store were associated with depression, while complaints related to difficulties with an executive basis were reported by those with elevated levels of anxiety. These findings provide some insights into the mechanisms that may underpin some of the difficulties reported by people with epilepsy and are consistent with the broader literature on subjective memory complaints in neurological patients.

It is unsurprising that high levels of comorbid anxiety and depression impact social function, and this has been observed in many patient groups.^28^, ^29^, ^30^ However, the mediation of these difficulties via the impact on memory function has received less attention in the literature, which has typically focused on the value of subjective memory complaints in differential diagnosis.

For example, Miebach et al. found unique patterns of cognitive complaints in patients with major depressive disorder (MDD) versus patients with mild cognitive impairment (MCI). Patients with MDD reported more complaints related to a lack of drive; for instance, attentional fluctuations and difficulties initiating tasks, while patients with MCI expressed extensive memory difficulties (memory for names, forgetfulness, and problems with prospective memory), executive functioning deficits, visuospatial difficulties, and dyscalculia.^31^

In our study, we found patients with elevated depression reported more complaints related to losses from the core store of memories; for instance, getting lost and forgetting autobiographical details. These complaints are not unique to those with epilepsy and depression. Disruptions in autobiographical and working memory are common symptoms of major depression.^32^ In severe cases, these symptoms are associated with a reduction in hippocampal volume and suppression of adult hippocampal neurogenesis.^33^, ^34^ These studies emphasize the complex biopsychosocial underpinnings of these complaints.^35^

Farina et al. suggested that depressed individuals find it more difficult to recall specific memories from their past due to executive functioning problems.^36^ In our study, it was the patients with elevated levels of anxiety who reported more difficulties with an executive basis than those with depression. Meltzer et al. found similar results in their study looking at reported executive functioning problems in a group of non‐demented older adults; individuals with high levels of anxiety reported more executive functioning difficulties.^37^ Attentional Control Theory postulates that anxiety disrupts aspects of executive functioning such as the inhibition of task‐irrelevant stimuli and the ability to shift attention.^38^ Essentially, anxiety impairs performance by reducing attentional focus. Disruption of attention is also evident in the hypervigilance that people with anxiety and low mood often experience when they begin to focus on minor everyday memory lapses, particularly in social settings. This hypervigilance can create a vicious cycle of increased anxiety, leading to further memory lapses and even greater levels of anxiety and hypervigilance.^1^

## 5 LIMITATIONS

This study focused on the nature of subjective memory complaints rather than psychometric measures of cognitive function. We have previously reported on the relationship between subjective memory complaints and performance on objective memory complaints^1^ and performance on objective memory tests and mood^39^, ^40^ in subsamples of the patients reported here. As outlined in the introduction, subjective complaints are not associated with objective performance in a wide range of cognitive domains^41^ and dissociate from measures of attention and working memory^9^ and verbal fluency and attention.^42^ Preliminary analysis of data from a subset of this group indicates that anxiety and low mood do not have a uniform impact on cognitive function assessed on psychometric tests.^39^

It is also important to recognize that our measures of anxiety and depression came from a simple screening measure – the Hospital Anxiety and Depression Scale. While the HADS generally has good validity in people with epilepsy^43^ it falls someway short of the gold standard of formal psychiatric evaluation and diagnosis. Further work would be required to validate these findings in a sample with a formal psychiatric diagnosis of generalized anxiety disorder and major depressive disorder.

Other features of the sample limit the generalizability of the results. Our sample came from a tertiary specialist service of people with epilepsy who had been specifically referred for a neuropsychological evaluation. This is not a service available to everyone with epilepsy, and these people may not represent the majority of people with epilepsy who are managed outside a specialist service. People with epilepsy referred to our service may have more severe epilepsy and be on higher doses of antiseizure medication, both of which may be biomarkers for greater underlying neurological dysfunction. It is therefore unclear how far we can generalize our findings to people with epilepsy whose seizures are well controlled on a single medication.

This study was conducted in the United Kingdom. We do not routinely collect data on the race and ethnicity of patients referred to our service in the United Kingdom. Education is compulsory in our system until the age of 18, and university level of education correlates more with age than ability, since only 3% of people had a university education in the 1960s compared to 40% in the 2020s. We appreciate that the very heterogeneous nature of the sample is a limitation of the study—these are unoperated patients with epilepsy who have been referred for a memory assessment, and those are the only two things they may have in common. A different study would be required to examine the impacts of race, ethnicity, education, socioeconomic status, and epilepsy‐related variables on subjective reports of memory dysfunction. We cannot ascribe any causality to the associations observed in our data, and it is likely that the relationships observed will be mediated by a variety of clinical and demographic variables that have not been included in our analyses. However, the advantage of this large heterogeneous group of people with epilepsy is that it does accurately reflect the caseload of referrals to a neuropsychology department, and, as such, provides useful data to help guide clinical practice. See clinical implications below.

While our findings highlight the importance of considering and addressing mood when presented with subjective memory complaints in the epilepsy clinic, mood is not the only explanatory factor for subjective complaints. Illness perceptions^10^ and general psychological distress^44^ are also highly correlated with subjective memory complaints.

Subjective memory complaints should not solely be interpreted as a reflection of poor mood; in some cases, they may represent something more sinister. In a recent study, patients reporting subjective memory decline (SMD) were found to show accelerated long‐term forgetting (ALF) with retention of verbal material. The authors suggest that ALF may underlie the memory complaints reported in SMD patients. They suggest that SMD represents a cognitive marker to reveal preclinical phases of neurodegenerative conditions such as Alzheimer's disease.^23^

### 5.1 Clinical implications

Understanding the nature and etiology of subjective memory complaints is an important aspect of epilepsy management. Our findings indicate that patients with high levels of anxiety and depression differ in both the nature and extent of subjective memory complaints. Clinicians should be alert to these signature subjective memory complaints reported in neurology clinics as they may be more indicative of anxiety and/or depression rather than a fundamental impairment in underlying memory processes. While the etiology of memory complaints in people with epilepsy is likely to be multifactorial, reflecting a complex interaction of organic and nonorganic factors in most cases, careful attention to the nature of patients' complaints may inform optimal treatment approaches. In a practical application, clinicians may consider probing more deeply about the nature of memory difficulties when they are raised in the clinic, either via semi‐structured interview questions or by using a validated measure of subjective memory complaints.^45^ Some developed specifically for people with epilepsy, such as the one used in this study, focus on how frequently people experience different types of problems, while others such as the MAC‐E^46^ or MMQ^47^ explore types of memory complaints in addition to the level of concern and use of compensation strategies. Information gathered from these measures may prompt further formal exploration of the impact of mood, particularly if difficulties in social aspects of function are reported. In settings where access to clinical neuropsychological and neuropsychiatric expertise is limited or subject to long waiting lists, these measures may help to prioritize referrals to the appropriate services. For neuropsychologists, these patterns may help in the interpretation, formulation, and feedback of the results from a formal assessment.

## CONFLICT OF INTEREST STATEMENT

None of the authors have any conflicts of interest to disclose in relation to this work.

## ETHICS STATEMENT

We confirm that we have read the Journal's position on issues involved in ethical publication and affirm that this report is consistent with those guidelines.

## Data Availability

The data that support the findings of this study are available on request from the corresponding author. The data are not publicly available due to privacy or ethical restrictions.

## APPENDIX A

### SUBJECTIVE MEMORY QUESTIONNAIRE

1. Forget where you have put something or lose things around the house?
2. Fail to recognize places that you are told you have been to often before?
3. Fail to remember a change in your daily routine, such as a change in the place where something is kept, or a change in the time something happens?
4. Have to go back and check whether you have done something you meant to do?
5. Forget that you were told something yesterday or a few days ago and maybe have to be reminded of it?
6. Let yourself ramble on and speak about unimportant or irrelevant things?
7. Have difficulty picking up a new skill, for example, finding it hard to learn a new game or to work some gadget after you have practised once or twice?
8. Find that a word is on the “tip of your tongue”: you know what it is but you cannot find it?
9. Forget important details of what you did or what happened to you the day before?
10. Forget important details about yourself, for example, date of birth or where your live?
11. Forget what you have just said to somebody, maybe saying “what was I talking about?”
12. Find it difficult to follow the thread of the story in a newspaper or magazine, and lose track of what it is about?
13. Forget to tell somebody something important, for example, forgetting to pass on a message or remind someone of something?
14. Get what someone has told you mixed up and confused?
15. Forget people's names?
16. Get lost or turn in the wrong direction on a journey, on a walk, or in a building you have been in only once or twice before?
17. Repeat to someone what you have told them, or ask them to same question twice?
18. Fail to recognize by sight a close relative?
19. Forget to take tablets?

Patients are asked to rate with which they experience these 19 different types of memory issues. Patients can choose the following responses: A = not at all, B = about once in the last 3 months, C = about once a month, D = about once a week, E = about once a day, and F = more than once a day.

The responses are scored on a Likert scale from 1 to 6 (A = 1, B = 2, and so on). The sum of the 19 items provides an overall SMQ score. Scores on the SMQ range from 19 (none of the difficulties are ever or rarely experienced) to 114 (all of the difficulties experienced more than once a day).
